# Supplementary material for: Predictive evolutionary modelling for influenza virus by site-based dynamics of mutations
Source: Nat Commun. 2024 Mar 21;15:2546. doi: 10.1038/s41467-024-46918-0 (PMC10958014; doi:10.1038/s41467-024-46918-0)
Supplement: Supplementary file 1 — Supplementary Information [file 41467_2024_46918_MOESM1_ESM.pdf]

Title: Predictive evolutionary modelling for influenza virus by site-based dynamics of mutations

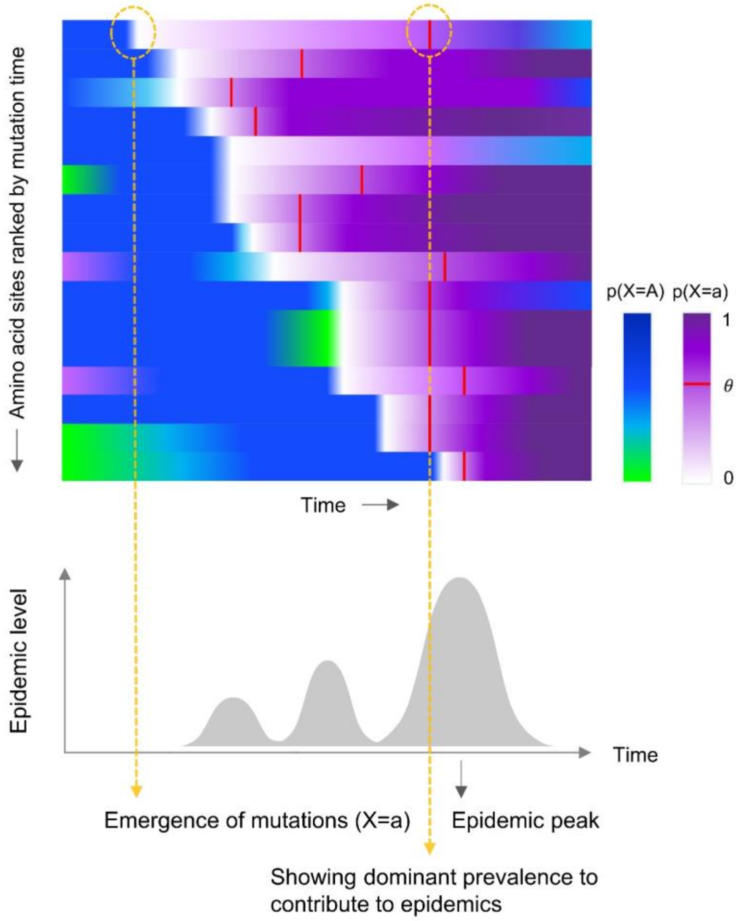

Supplementary Fig. 1 Schematic plot of the relationship between mutation prevalence dynamics in genome and population epidemics. Each color represents the dynamic change of a mutations at a genomic site (by row). Mutations with selective advantage emerge and lead to viral fitness at varying paces. For example, in the first row of the upper panel, P($X = a$), the mutation prevalence of a residue *a* at a codon position *X* gradually increases from 0 (white) to 1 (purple). θ is the mutation prevalence threshold indicating that the substitution is detected to be significantly associated with the rise of epidemics level. P($X = A$) is the prevalence of prior residue *A* at position *X*, ranges from 0 (green) to 1 (blue). The proposed method draws information from the mutation transition time to model the dynamics of evolution.

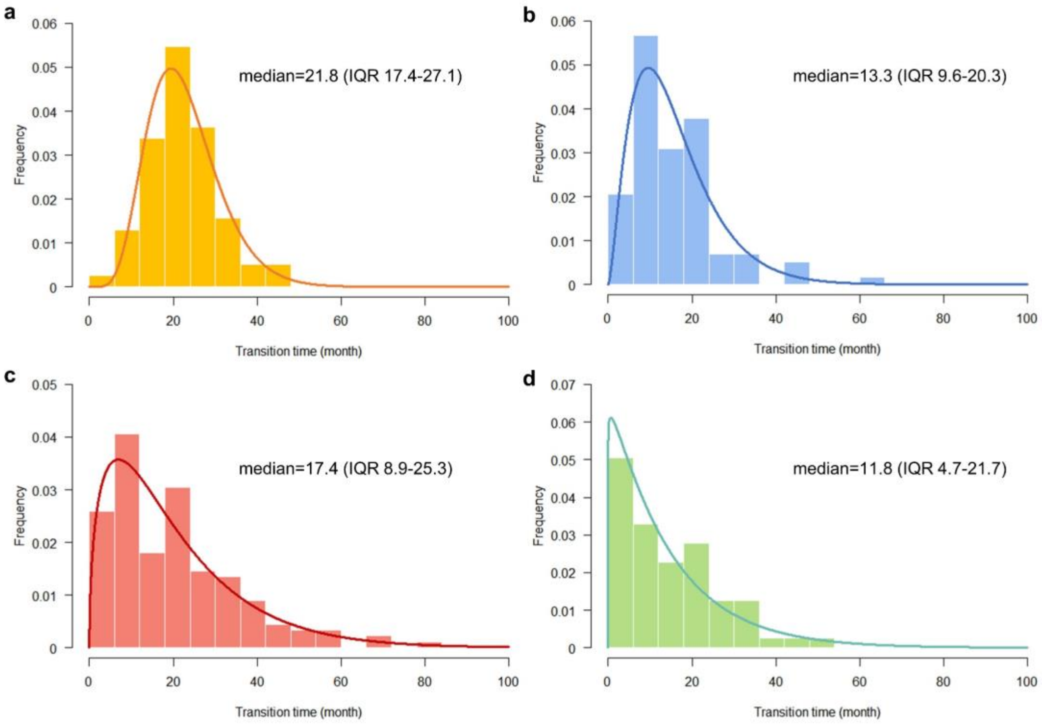

Supplementary Fig. 2 Empirical probability distribution of transition time $\boldsymbol{\tau}$. a, pH1N1 HA. **b**, pH1N1 NA. **c**, H3N2, HA. **d**, H3N2 NA. The transition time $\boldsymbol{\tau}$ of mutation from emergence to an influential threshold θ for population epidemics exhibits gamma-like distribution. IQR: the interquartile range Q1 to Q3.

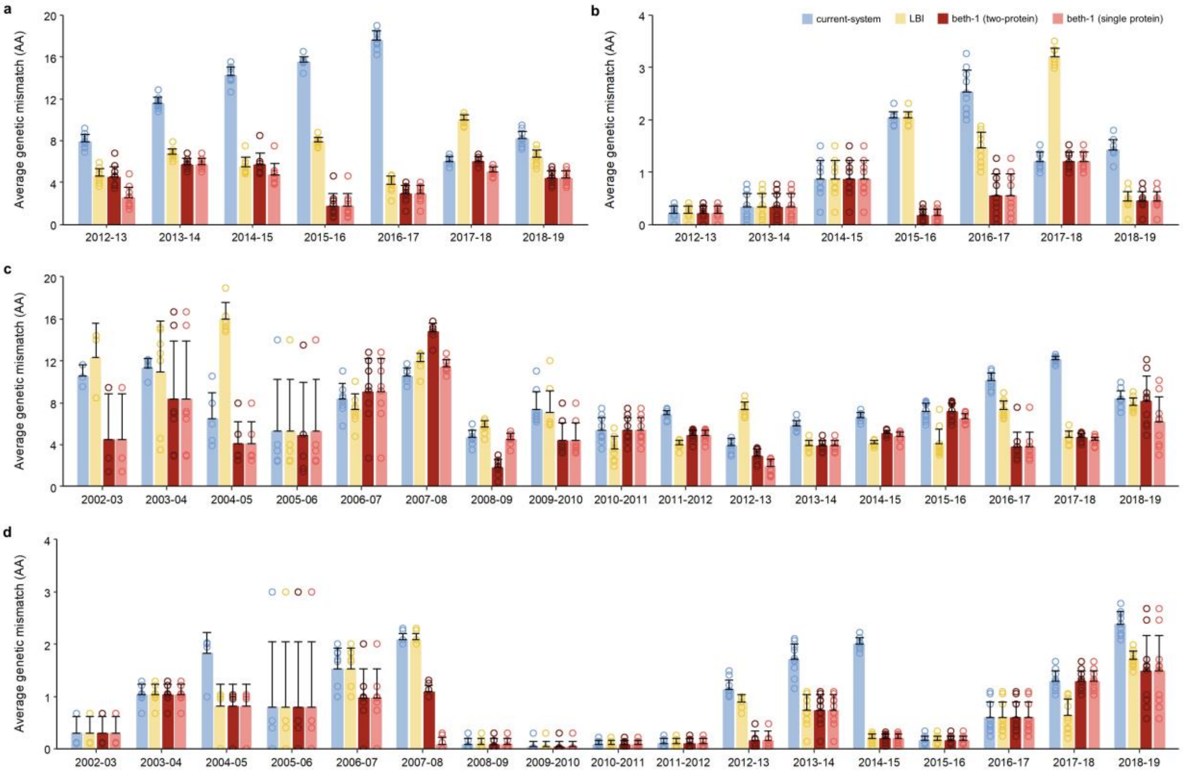

Supplementary Fig. 3 Prediction accuracy of alternative methods for NA evolution in the retrospective data by season.

a, pH1N1, NA full sequence (469 codons). **b**, pH1N1, NA epitopes (30 sites). **c**, H3N2, NA full sequence (469 codons). **d**, H3N2, NA epitopes (12 sites). Prediction accuracy is assessed by the average amino acids mismatch (Y-axis) of the predicted strains against circulating viruses in ten geographical regions in the respective epidemic seasons (X-axis). Error bar: standard deviation of average genetic mismatch by region in a given season.

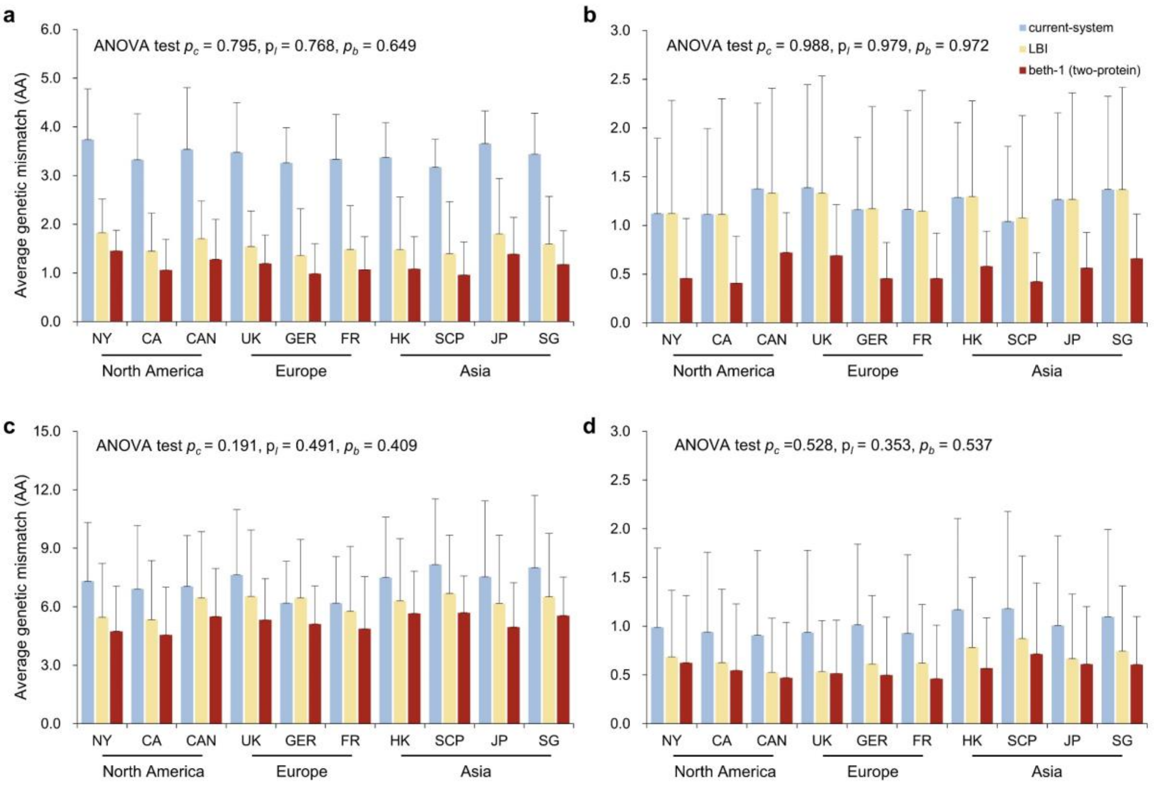

Supplementary Fig. 4 Prediction accuracy on epitopes, breakdown by geographical region. a, pH1N1, HA epitopes. **b**, pH1N1, NA epitopes. **c**, H3N2, HA epitopes. **d**, H3N2 NA epitopes. p_c: Two-sided *p*-value of ANOVA test on equality of genetic mismatch across three continents by the current-system; p_l: by LBI; p_b: by beth-1 (two-protein). The sample size n = 21 independent data in North America, n = 21 independent data in Europe and n = 28 independent data in Asia (panel **a**, **b**); n = 51 independent data in North America, n = 51 independent data in Europe and n = 68 independent data in Asia (panel **c**, **d**). Error bar: standard deviation of the average genetic mismatch by season in a given region. No significant difference in genetic mismatch is observed across continents using either prediction models.

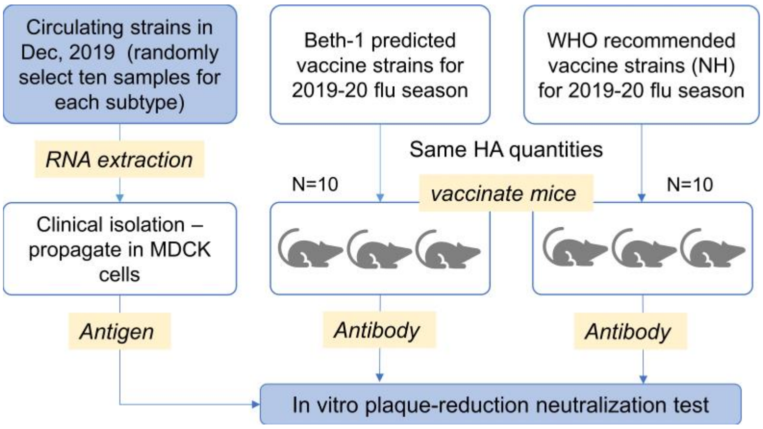

Supplementary Fig. 5 Experimental design of in vivo experiment. Batches of female BALB/c mice aged at 6-8 weeks were immunized intraperitoneally by recombinant influenza virus with the predicted HA and NA genes by the current-system and beth-1 were. The levels of neutralizing antibodies in antisera were determined by PRNT using clinical isolates of the pH1N1 or H3 viruses.

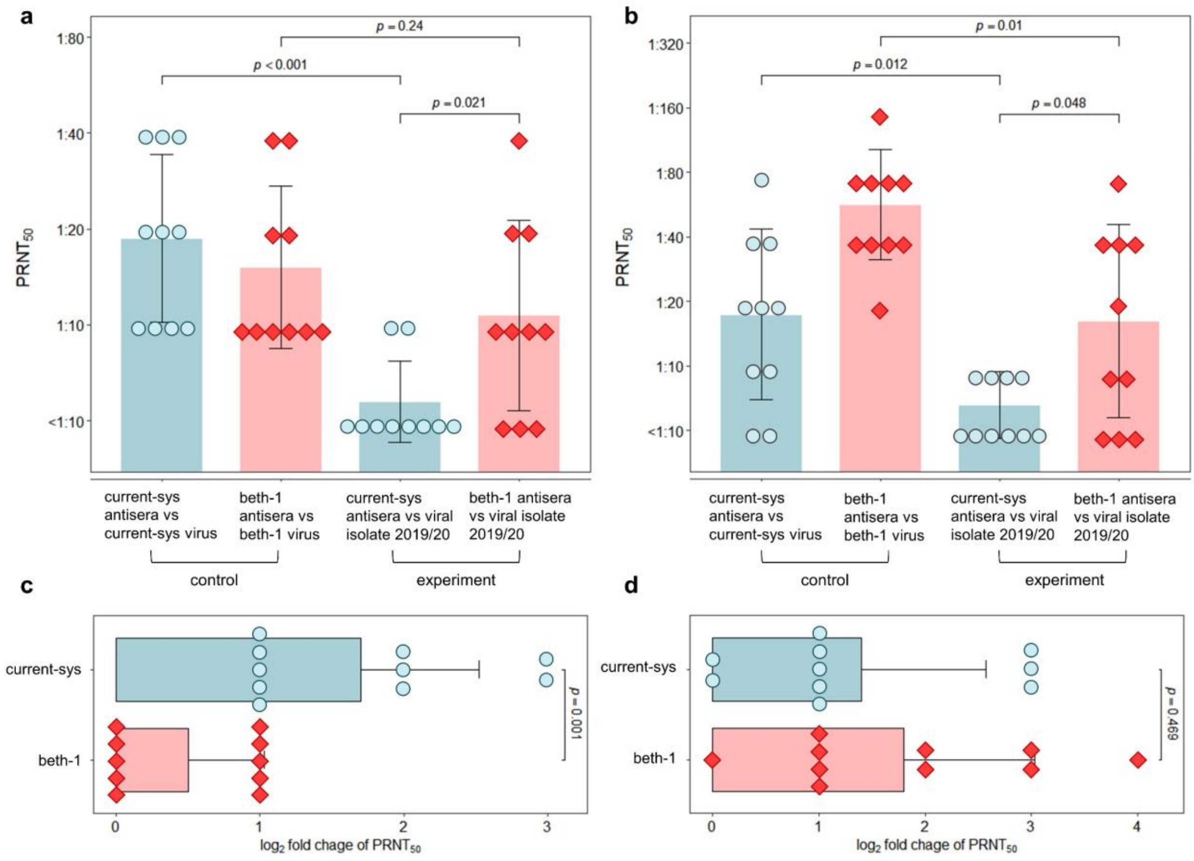

Supplementary Fig. 6 Neutralization of antibodies induced by the predicted strains for the 2019/20 season in Hong Kong. a, neutralization for pH1N1 virus. **b**, neutralization for H3N2 virus. **c**, pH1N1, titer fold-change. **d**, H3N2, titer fold-change. The neutralizing effect of antibodies elicited by predicted strains in mouse model was evaluated by $PRNT_{50}$ titer against virus isolates and compared to those elicited by strains predicted by the current system. Control groups: antibodies elicited by predicted strain neutralizing the predicted strain. Experimental group: antibodies elicited by predicted strains neutralizing viral isolates in Hong Kong 2019/20. N = 10 mice for both control group and experiment group. Fold-change: the ratio of titer of control group to titer of experimental group. Beth-1 predicted strains (red color) generated neutralization significantly higher (two-sided *t*-test *p*-value = 0.001) for the pH1N1 and non-inferior for the H3N2 (two-sided *t*-test *p*-value = 0.469) comparing to current-system strains (green color).

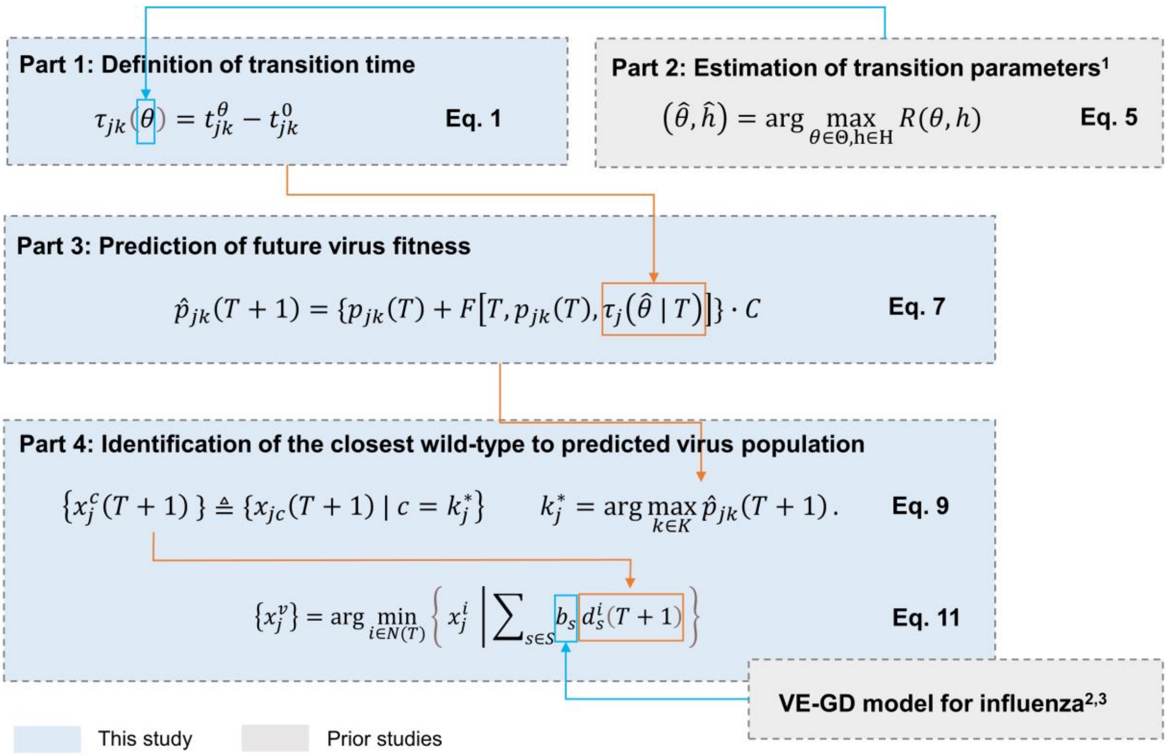

Supplementary Fig. 7 beth-1 method flowchart. The computational framework is composed of 4 parts, and the figure illustrates the relationship between the parts.

Supplementary Table 1. Effective mutations and predictor codon sites. Effective mutation sites (EMs) emerged in each season are listed. **Boldface**: epitope sites. <u>Underline</u>: predictor codon sites. The codon sites of pH1N1 are in H3 numbering.

Flu season	HA protein	NA protein
Subtype: pH1N1		
2011/12	**<u>188</u>**, 453	241, 369
2012/13	2, 92, 146, 200, 285, 501	44, 200, 232
2013/14	237	-
2014/15	**<u>166</u>**, 259, 271	34, 40, 321, 432
2015/16	7	13, **<u>264</u>**, **<u>270</u>**, 314
2016/17	**<u>165</u>**, 219	-
2017/18	**<u>82</u>**, 218, 297	188, **<u>449</u>**
2018/19	**<u>167</u>**	77, 81
2019/20	186	51, 74, 416, 452
2020/21	133, 134, **<u>159</u>**, **<u>164</u>**, **<u>190</u>**, **<u>192</u>**, 253, 262, 508	19, 52, 66, 222, **<u>389</u>**
2021/22	<u>169</u>, 205, 368	432
2022/23	101	469
Subtype: H3N2		
2002/03	25, 75, 83, **<u>155</u>**, **<u>156</u>**, 183, **<u>186</u>**, 202, 222, **309**, 500, 530	30, 42, 143, 216, 332, 385
2003/04	479	-
2004/05	**<u>126</u>**, **<u>138</u>**, **<u>145</u>**, **<u>159</u>**, **<u>189</u>**, **227**	18, 40, 93, 172, **<u>199</u>**, 265, 399, 432, 437
2005/06	**<u>193</u>**, **226**, 326, 361, 484	**<u>221</u>**
2006/07	6, **<u>157</u>**, 375, 450	4, 5, 43, 194, 310, 315, **<u>370</u>**
2007/08	**<u>142</u>**	86, **<u>150</u>**, 151, 296, 335
2009/10	-	-
2010/11	**62**, **<u>158</u>**, **213**	-
2011/12	**53**, **94**, **212**, **230**, **280**	210, 367, 369, 464
2012/13	**45**, **48**, **<u>198</u>**, 223, **278**, **312**	81, 402
2013/14	-	-
2014/15	**<u>128</u>**	392
2015/16	3, **<u>160</u>**, **311**	- -
2016/17	**171**, 406, 489	245, 247, 339, 380, 468
2017/18	**261**	-
2018/19	**121**, **<u>135</u>**	**<u>220</u>**, 303, **<u>329</u>**
2019/20	**<u>137</u>**, 529	315, **<u>344</u>**
2020/21	-	469
2021/22	**83**, **<u>131</u>**, **<u>164</u>**, **<u>186</u>**, **<u>190</u>**, 195, 522	62, 346, 463, 465
2022/23	104, **276**	-

Supplementary Table 2. Sequence sample size. Complete genetic sequences of influenza virus pH1N1 and H3N2 were retrieved from the Global Initiative on Sharing all Influenza Data (GISAID). The total number of sequences used in analysis is 50,285 for hemagglutinin (HA) and 45,297 for Neuraminidase (NA).

Flu season	pH1N1		H3N2	
	HA	NA	HA	NA
1999/00	-	-	87	87
2000/01	-	-	25	23
2001/02	-	-	112	110
2002/03	-	-	52	50
2003/04	-	-	236	237
2004/05	-	-	164	161
2005/06	-	-	73	73
2006/07	-	-	86	84
2007/08	-	-	88	85
2008/09	-	-	434	426
2009/10	1817	922	129	128
2010/11	226	167	325	295
2011/12	51	47	546	454
2012/13	184	163	454	305
2013/14	461	448	626	508
2014/15	289	288	1733	1329
2015/16	1335	1310	852	789
2016/17	551	551	2907	2477
2017/18	1220	1122	2179	1670
2018/19	2063	1714	2548	2190
2019/20	1566	1193	1875	1671
2020/21[a]	122	111	494	469
2021/22[a]	1358	1360	15476	15074
2022/23	1949	1864	5592	5342
**Sum**	**13,192**	**11,260**	**37,093**	**34,037**

[a] In these periods, all available sequences in the Northern Hemisphere (NH) were retrieved. Other than these epidemics, all available sequences from ten geographical regions in NH were retrieved, include North America (New York State, California State, Canada), Europe (United Kingdom, Germany, France), and Asia (Hong Kong SAR, South China provinces, Japan, Singapore).

Supplementary Table 3. Summary table of predicted strains

Flu season	WHO recommended vaccine strains[a]	beth-1 strain (two-protein model) (sequence collection time)	Answer strain[b]
Subtype: pH1N1			
2012/13	A/California/07/2009	A/India/Nsk12388/2012 (2012-01-19)	A/India/2080/2012 – like virus
2013/14	A/California/07/2009	A/Singapore/GP325/2013 (2013-02-07)	A/Hong Kong/6015/2013 – like virus
2014/15	A/California/07/2009	A/Singapore/KK107/2014 (2014-01-06)	A/Norway/2432/2014 – like virus
2015/16	A/California/07/2009	A/India/P153017/2015 (2015-02-23)	A/Hong Kong/15587/2015 – like virus
2016/17	A/California/07/2009	A/Prachuapkhirikhan/42/2016 (2016-02-29)	A/Singapore/SGH0769/2016 – like virus
2017/18	A/Michigan/45/2015	A/Phra Nakhon Si Ayutthaya/396/2016 (2016-11-01)	A/Oman/5640/2017 – like virus
2018/19	A/Michigan/45/2015	A/Hong Kong/4989/2017 (2017-12-17)	A/Lipetsk/1V/2018 – like virus
2019/20[c]	A/Brisbane/02/2018	A/Hong Kong/98/2019 (2019-01-01)<br>A/Hong Kong/2655/2019 (2019-06-18)	A/Bahrain/578/2019 – like virus
2020/21	A/Guangdong-Maonan/SWL1536/2019	A/Hong Kong/4380/2019 (2019-11-27)	A/India/PUN-NIV323483/2021 – like virus
2021/22	A/Victoria/2570/2019	A/Nagasaki/8/2020 (2020-10-27)	A/Netherlands/10201/2021 – like virus
2022/23	A/Victoria/2570/2019	A/Bangladesh/3210910005/2021 (2021-09-07)	A/Denmark/3091/2022 – like virus
Subtype: H3N2			
2002/03	A/Moscow/10/99	A/Hong Kong/CUHK5250/2002 (2002-01-10)	A/Denmark/32/03 – like virus
2003/04	A/Moscow/10/99	A/Hong Kong/CUHK5316/2003 (2003-01-10)	A/New York/54/2003 – like virus
2004/05	A/Fujian/411/2002	A/Singapore/NHRC0012/2003 (2003-10-13)	A/Hong Kong/CUHK10026/2005 – like virus
2005/06	A/California/07/2004	A/Guangdong/03/2005 (2005-01-01)	A/Illinois/NHRC0002/2005 – like virus
2006/07	A/Wisconsin/67/2005	A/Thailand/CU259/2006 (2006-01-01)	A/Singapore/68Q/2007 – like virus
2007/08	A/Wisconsin/67/2005	A/Beijing/Xicheng1272/2006 (2006-12-11)	A/Florida/UR07-0101/2008 – like virus
2008/09	A/Brisbane/10/2007	A/Thailand/CU379/2008 (2008-01-01)	A/California/VRDL375/2009 – like virus
2009/10	A/Brisbane/10/2007	A/Thailand/CU379/2008 (2008-01-01)	A/Florida/26/2009 – like virus
2010/11	A/Perth/16/2009	A/Hong Kong/H090-783-V10/2009 (2009-08-07)	A/Kentucky/12/2010 – like virus
2011/12	A/Perth/16/2009	A/Singapore/C2011.027V/2011 (2011-01-10)	A/Montana/08/2011 – like virus
2012/13	A/Victoria/361/2011	A/Hunan-Kifu/1166/2012 (2012-01-01)	A/Arizona/09/2012 – like virus
2013/14	A/Victoria/361/2011	A/Hong Kong/8147/2012 (2012-06-26)	A/South Carolina/03/2014 – like virus
2014/15	A/Texas/50/2012	A/Singapore/H2013.403/2013 (2013-05-27)	A/Texas/38/2014 – like virus
2015/16	A/Switzerland/9715293/2013	A/Shandong-Linzi/323/2014 (2014-12-01)	A/Colorado/15/2015 – like virus
2016/17	A/Hong Kong/4801/2014	A/Chiang Mai/54/2016 (2016-02-13)	A/Singapore/TT0348/2017 – like virus
2017/18	A/Hong Kong/4801/2014	A/Singapore/KK0164/2017 (2017-02-20)	A/Pennsylvania/263/2017 – like virus
2018/19	A/Singapore/INFIMH-16-0019/2016	A/Wuhan/2785/2018 (2018-02-01)	A/Kyiv/537/2018 – like virus
2019/20	A/Kansas/14/2017	A/Hong Kong/2926/2018 (2018-11-26)<br>A/Hong Kong/2678/2019 (2019-06-18)	A/Hungary/9/2020 – like virus
2020/21	A/Hong Kong/2671/2019	A/Hong Kong/4435/2019 (2019-11-29)	A/Michigan/03/2021 – like virus
2021/22	A/Cambodia/e0826360/2020	A/Bangladesh/0004/2020 (2020-09-06)	A/Michigan/08/2021 – like virus
2022/23	A/Darwin/6/2021	A/Arsal/5773/2021 (2021-09-13)	A/England/224040474/2022 – like virus

[a] The WHO recommended vaccine for Northern Hemisphere were collected from influenza vaccine sheet of WHO, accessed via https://www.who.int/teams/global-influenza-programme/vaccines/who-recommendations, and the sequences were retrieved from GISAID [25].

[b] The answer strain is the closest wild-type strain to the consensus strain obtained from the observed circulating virus in the target season.

[c] Shaded rows: Seasons in prospective study.

Supplementary Table 4. Summary table of predicted strains by the LBI and beth-1 (single protein)

Flu season	beth-1 vaccine strain (HA)	beth-1 vaccine strain (NA)	LBI (HA)[a]	LBI (NA)
Subtype: pH1N1				
2012/13	A/India/Nsk12388/2012	A/Srinigar/827/2011	A/Florida/19/2011	A/Florida/27/2011
2013/14	A/Singapore/GP325/2013	A/Singapore/GP325/2013	A/Taiwan/989/2012	A/Lithuania/5509/2013
2014/15	A/Singapore/KK107/2014	A/Singapore/KK1064/2013	A/Haiti/2036/2013	A/YOKOHAMA/162/2013
2015/16	A/India/3388/2015	A/India/P153017/2015	A/Berlin/87/2014	A/Trencin/298/2015
2016/17	A/Prachuapkhirikhan/42/2016	A/Prachuapkhirikhan/42/2016	A/Kyrgyzstan/71/2015	A/Wisconsin/21/2016
2017/18	A/Phra_Nakhon_Si_Ayutthaya/396/2016	A/Singapore/TT0169/2017	A/Ohio/07/2017	A/Shanghai-Minxing/SWL113/2017
2018/19	A/Singapore/GP0014/2018	A/Singapore/EN0138/2018	A/Washington/299/2017	A/Jeju/836/2017
Subtype: H3N2				
2002/03	A/Hong Kong/CUHK5250/2002	A/Hong Kong/CUHK5250/2002	A/New York/92/2002	A/Texas/NHRC0001/2002
2003/04	A/Hong Kong/CUHK5316/2003	A/Hong Kong/CUHK6383/2003	A/Hong Kong/CUHK33047/2002	A/Genoa/1/2002
2004/05	A/Hong Kong/CUHK74438/2003	A/Hong Kong/HKU1/2004	A/Thailand/Siriraj-02/2003	A/New Jersey/NHRC0001/2003
2005/06	A/Guangdong/04/2005	A/Hong Kong/CUHK7047/2005	A/New York/469/2004	A/South Carolina/NHRC0001/2005
2006/07	A/Thailand/CU259/2006	A/Thailand/CU259/2006	A/New York/1001/2005	A/New York/1012/2006
2007/08	A/Beijing/Xicheng1272/2006	A/Hong Kong/CUHK53005/2006	A/Vietnam/19/2007	A/Honduras/45/2006
2008/09	A/Thailand/CU379/2008	A/Thailand/CU379/2008	A/Singapore/65/2007	A/Louisiana/05/2008
2009/10	A/Hong Kong/40/2009	A/Singapore/619/2008	A/Korea/WRAIR1391P/2009	A/North Dakota/02/2008
2010/11	A/Hong Kong/H090-689-V2	A/SURAT_THANI/116/2010	A/Oregon/08/2009	A/Pennsylvania/02/2010
2011/12	A/Singapore/KK114/2011	A/Singapore/H2011.140C/2011	A/Pennsylvania/10/2010	A/Ontario/P21548/2010
2012/13	A/Hunan-Kifu/1166/2012	A/China/89/2012	A/Singapore/KK219/2011	A/Paris/2100/2011
2013/14	A/Delhi/764/2013	A/Hangzhou/A155/2013	A/Helsinki/942/2013	A/Houston/JMM 158/2013
2014/15	A/Anhui-Yaohai/1704/2013	A/Singapore/GP415/2014	A/Florida/21/2013	A/Norway/3303/2013
2015/16	A/Shaanxi-Baota/1661/2014	A/China/60376/2014	A/OSAKA/63/2014	A/Tennessee/16/2014
2016/17	A/Shanxi-Yaodou/1327/2016	A/Singapore/INFTT-16-0224/2016	A/Ontario/RV2425/2015	A/Mexico/92/2016
2017/18	A/Singapore/GP0269/2017	A/Singapore/TT0096/2017	A/Singapore/MOH0107/2016	A/Champagne Ardenne/628/2017
2018/19	A/Wuhan/2785/2018	A/Singapore/TT0256/2018	A/Zhejiang-Yongkang/1978/2017	A/Ireland/61097/2017

[a] When the highest LBI score corresponds to multiple strains in a season, one of the strains is randomly picked.

Supplementary Table 5. Summary of prediction performance in retroactive validation by alternative methods for the HA and NA genes of pH1N1 and H3N2

Subtype	Prediction seasons	Gene	Average number of amino acids mismatch[a,b] (SD)						
			current-system	beth-1 (HA+NA)	answer strain	LBI (HA)	beth-1 (HA)	LBI (NA)	beth-1 (NA)
pH1N1	2012/13 - 2018/19	HA full-length	12.8 (4.4)	5.1 (2.8)	3.0 (1.2)	5.7 (2.5)	5.1 (2.8)	-	-
		NA full-length	11.6 (4.4)	4.4 (1.6)	2.9 (0.9)	-	-	6.4 (2.1)	3.9 (1.5)
		HA epitope	3.4 (0.8)	1.2 (0.6)	0.7 (0.4)	1.6 (0.9)	1.2 (0.6)	-	-
		NA epitope	1.2 (0.8)	0.5 (0.4)	0.4 (0.2)	-	-	1.2 (1.1)	0.5 (0.4)
H3N2	2002/03 - 2018/19	HA full-length	11.7 (5.1)	7.5 (2.2)	5.4 (1.7)	9.5 (4.7)	7.5 (2.2)	-	-
		NA full-length	7.7 (2.5)	5.7 (3.0)	4.3 (2.2)	-	-	7.3 (3.6)	5.3 (2.3)
		HA epitope	7.7 (3.4)	5.1 (1.7)	3.5 (1.1)	6.1 (2.9)	5.1 (1.7)	-	-
		NA epitope	1.0 (0.8)	0.6 (0.5)	0.4 (0.4)	-	-	0.7 (0.6)	0.5 (0.5)

[a] Average amino acids mismatch calculated from pair-wise mismatch between circulating strains against vaccine strain. Epidemic seasons predicted: for pH1N1, 7 seasons from 2012/13 to 2018/19; and for H3N2, 17 seasons from 2002/03 to 2018/19. Season-to-season breakdown is in **Extended Data Table 6-9**.

[b] Ten geographical regions evaluated in Northern Hemisphere, covering North America (New York State, California State, Canada), Europe (United Kingdom, Germany, France), and Asia (Hong Kong SAR, South China provinces, Japan, Singapore). Genetic mismatch breakdown by geographical region is in **Extended Data Table 10-13**.

Supplementary Table 6. Prediction performance in retrospective data by epidemic season for HA of pH1N1

Epidemic season (sample size)	Average number of mismatch on HA sequence (out of 566 codons) (amino acids, AAs)				Average number of mismatch[a] on HA epitope (out of 50 sites) (AAs)			
	current-system (SD)	beth-1 (HA+NA) (SD)	LBI (HA) (SD)	beth-1 (HA) (SD)	current-system (SD)	beth-1 (HA+NA) (SD)	LBI (HA) (SD)	beth-1 (HA) (SD)
2012/13 (N=184)	13.0 (0.6)	10.4 (1.1)	10.2 (1.1)	10.4 (1.1)	3.0 (0.3)	1.0 (0.3)	2.8 (0.3)	1.0 (0.3)
2013/14 (N=461)	12.9 (0.6)	4.3 (0.4)	5.4 (0.4)	4.3 (0.4)	3.2 (0.2)	1.2 (0.2)	1.2 (0.2)	1.2 (0.2)
2014/15 (N=289)	14.1 (1.1)	2.2 (1.1)	2.2 (1.1)	2.2 (1.1)	3.4 (0.4)	0.4 (0.4)	0.4 (0.4)	0.4 (0.4)
2015/16 (N=1,335)	16.8 (0.5)	3.3 (1.1)	4.9 (0.6)	3.3 (1.1)	4.0 (0.2)	1.0 (0.2)	1.0 (0.2)	1.0 (0.2)
2016/17 (N=551)	18.6 (0.8)	2.9 (0.6)	3.9 (0.6)	2.9 (0.6)	5.0 (0.6)	1.1 (0.5)	1.1 (0.5)	1.1 (0.5)
2017/18 (N=1,220)	5.9 (0.9)	6.9 (0.9)	6.9 (0.9)	6.9 (0.9)	2.4 (0.2)	2.4 (0.2)	2.4 (0.2)	2.4 (0.2)
2018/19 (N=2,063)	8.4 (0.9)	5.4 (0.9)	6.4 (0.9)	5.4 (0.9)	3.2 (0.3)	1.2 (0.3)	2.2 (0.3)	1.2 (0.3)

[a] Average amino acids mismatch against all available circulating strains in respective epidemic season in ten geographical regions.

Supplementary Table 7. Prediction performance in retrospective data by epidemic season for NA of pH1N1

Epidemic season (sample size)	Average number of mismatch on HA sequence (out of 469 codons) (AAs)				Average number of mismatch[a] on HA epitope (out of 30 sites) (AAs)			
	current-system (SD)	beth-1 (HA+NA) (SD)	LBI (NA) (SD)	beth-1 (NA) (SD)	current-system (SD)	beth-1 (HA+NA) (SD)	LBI (NA) (SD)	beth-1 (NA) (SD)
2012/13 (N=163)	7.9 (0.7)	4.5 (1.1)	4.6 (0.7)	2.5 (1.0)	0.2 (0.1)	0.2 (0.1)	0.2 (0.1)	0.2 (0.1)
2013/14 (N=448)	11.6 (0.6)	5.8 (0.5)	6.7 (0.5)	5.8 (0.5)	0.3 (0.3)	0.3 (0.3)	0.3 (0.3)	0.3 (0.3)
2014/15 (N=288)	14.2 (0.8)	5.7 (1.1)	5.6 (0.8)	4.7 (1.1)	0.9 (0.4)	0.9 (0.4)	0.9 (0.4)	0.9 (0.4)
2015/16 (N=1,310)	15.5 (0.5)	1.7 (1.2)	7.8 (0.5)	1.7 (1.2)	2.0 (0.1)	0.2 (0.1)	2.0 (0.1)	0.2 (0.1)
2016/17 (N=551)	17.6 (0.8)	2.9 (0.8)	3.8 (0.7)	2.9 (0.8)	2.5 (0.4)	0.5 (0.4)	1.5 (0.3)	0.5 (0.4)
2017/18 (N=1,122)	6.0 (0.5)	6.0 (0.5)	9.9 (0.5)	5.0 (0.5)	1.2 (0.2)	1.2 (0.2)	3.2 (0.2)	1.2 (0.2)
2018/19 (N=1,714)	8.1 (0.8)	4.5 (0.6)	6.4 (0.7)	4.5 (0.6)	1.4 (0.2)	0.4 (0.2)	0.4 (0.2)	0.4 (0.2)

[a] Average amino acids mismatch against all available circulating strains in respective epidemic season in ten geographical regions.

Supplementary Table 8. Prediction performance in retrospective data by epidemic season for HA of H3N2

Epidemic season (sample size)	Average mismatch on HA sequence (out of 566 codons) (AAs)				Average mismatch[a] on HA epitope (out of 131 sites) (AAs)			
	current-system (SD)	beth-1 (HA+NA) (SD)	LBI (HA) (SD)	beth-1 (HA) (SD)	current-system (SD)	beth-1 (HA+NA) (SD)	LBI (HA) (SD)	beth-1 (HA) (SD)
2002/03 (N=52)	23.3 (2.9)	9.0 (1.5)	18.1 (4.1)	9.0 (1.5)	14.6 (1.9)	5.0 (0.3)	8.9 (2.0)	5.0 (0.3)
2003/04 (N=236)	25.1 (0.8)	4.7 (1.4)	4.8 (1.3)	4.7 (1.4)	16.5 (0.8)	3.2 (1.0)	4.2 (1.0)	3.2 (1.0)
2004/05 (N=164)	11.8 (3.5)	6.7 (3.8)	6.8 (3.8)	6.7 (3.8)	7.9 (2.4)	4.5 (2.5)	4.6 (2.4)	4.5 (2.5)
2005/06 (N=73)	10.3 (4.7)	6.7 (6.1)	7.5 (4.7)	6.7 (6.1)	6.3 (2.7)	3.1 (3.0)	3.5 (2.6)	3.1 (3.0)
2006/07 (N=86)	10.7 (2.7)	7.4 (2.4)	6.9 (2.9)	7.4 (2.4)	6.3 (1.4)	4.6 (1.3)	3.4 (1.5)	4.6 (1.3)
2007/08 (N=88)	11.0 (0.7)	9.1 (0.8)	7.9 (0.7)	9.1 (0.8)	6.6 (0.5)	5.6 (0.5)	5.4 (0.6)	5.6 (0.5)
2008/09 (N=434)	8.3 (1.7)	4.3 (1.7)	6.3 (1.7)	4.3 (1.7)	4.6 (1.5)	3.6 (1.6)	4.6 (1.5)	3.6 (1.6)
2009/10 (N=129)	11.7 (0.6)	7.7 (0.6)	17.6 (0.6)	7.7 (0.6)	7.6 (0.6)	6.6 (0.6)	10.6 (0.6)	6.6 (0.6)
2010/11 (N=325)	9.5 (0.7)	9.4 (0.8)	9.0 (0.7)	9.4 (0.8)	7.9 (0.6)	7.9 (0.7)	7.4 (0.6)	7.9 (0.7)
2011/12 (N=546)	11.0 (0.7)	10.1 (1.8)	7.2 (0.8)	10.1 (1.8)	8.6 (0.6)	7.7 (1.6)	4.8 (0.7)	7.7 (1.6)
2012/13 (N=454)	6.9 (0.8)	4.6 (1.1)	5.9 (0.8)	4.6 (1.1)	3.8 (0.7)	2.3 (0.8)	3.8 (0.7)	2.3 (0.8)
2013/14 (N=626)	9.3 (0.9)	5.5 (0.8)	18.8 (1)	5.5 (0.8)	5.2 (0.5)	3.3 (0.5)	13.8 (0.3)	3.3 (0.5)
2014/15 (N=1,733)	13.1 (0.5)	9.5 (0.8)	10.3 (0.7)	9.5 (0.8)	9.6 (0.3)	6.0 (0.5)	6.0 (0.5)	6.0 (0.5)
2015/16 (N=852)	11.5 (2.6)	10.9 (2.2)	5.8 (1.2)	10.9 (2.2)	7.3 (1.6)	7.3 (1.6)	3.8 (1.1)	7.3 (1.6)
2016/17 (N=2,907)	6.3 (0.8)	5.8 (1.1)	5.8 (1.1)	5.8 (1.1)	4.3 (0.8)	4.1 (0.9)	4.1 (0.9)	4.1 (0.9)
2017/18 (N=2,179)	6.8 (1.4)	6.0 (2.8)	7.6 (0.4)	6.0 (2.8)	5.4 (0.9)	4.7 (2.4)	5.6 (0.5)	4.7 (2.4)
2018/19 (N=2,548)	12.0 (2.6)	10.4 (3.3)	15.1 (2.6)	10.4 (3.3)	7.4 (1.2)	6.7 (1.9)	8.7 (2.4)	6.7 (1.9)

[a] Average amino acids mismatch against all available circulating strains in respective epidemic season, averaging the mismatch in ten geographical regions.

Supplementary Table 9. Prediction performance in retrospective data by epidemic season for NA of H3N2

Epidemic season (sample size)	Average number of mismatch on NA sequence (out of 469 codons) (AAs)				Average number of mismatch[a] on NA epitope (out of 12 sites) (AAs)			
	current-system (SD)	beth-1 (HA+NA) (SD)	LBI (NA) (SD)	beth-1 (NA) (SD)	current-system (SD)	beth-1 (HA+NA) (SD)	LBI (NA) (SD)	beth-1 (NA) (SD)
2002/03 (N=52)	10.5 (1.1)	4.5 (4.3)	12.3 (3.3)	4.5 (4.3)	0.3 (0.3)	0.3 (0.3)	0.3 (0.3)	0.3 (0.3)
2003/04 (N=236)	11.3 (1.0)	8.4 (5.6)	10.9 (4.9)	8.4 (5.6)	1.0 (0.2)	1.0 (0.2)	1.0 (0.2)	1.0 (0.2)
2004/05 (N=164)	6.4 (2.5)	4.1 (2.1)	16.0 (1.6)	4.1 (2.1)	1.8 (0.4)	0.8 (0.4)	0.8 (0.4)	0.8 (0.4)
2005/06 (N=73)	5.3 (4.9)	4.9 (5.0)	5.3 (4.9)	5.3 (4.9)	0.8 (1.3)	0.8 (1.3)	0.8 (1.3)	0.8 (1.3)
2006/07 (N=86)	8.3 (1.5)	9.0 (3.2)	7.3 (1.5)	9.0 (3.2)	1.5 (0.4)	1.0 (0.5)	1.5 (0.4)	1.0 (0.5)
2007/08 (N=88)	10.6 (0.7)	14.8 (0.8)	11.9 (0.9)	10.5 (0.7)	2.1 (0.1)	1.1 (0.1)	2.1 (0.1)	0.1 (0.1)
2008/09 (N=434)	4.7 (0.7)	1.8 (0.8)	5.6 (0.6)	1.8 (0.8)	0.1 (0.1)	0.1 (0.1)	0.1 (0.1)	0.1 (0.1)
2009/10 (N=129)	7.4 (1.6)	4.4 (1.6)	7.0 (2.2)	4.4 (1.6)	0.0 (0.1)	0.0 (0.1)	0.0 (0.1)	0.0 (0.1)
2010/11 (N=325)	5.4 (1.2)	5.4 (1.2)	3.6 (1.1)	5.4 (1.2)	0.1 (0.1)	0.1 (0.1)	0.1 (0.1)	0.1 (0.1)
2011/12 (N=546)	6.9 (0.4)	4.9 (0.5)	3.9 (0.5)	4.9 (0.5)	0.1 (0.1)	0.1 (0.1)	0.1 (0.1)	0.1 (0.1)
2012/13 (N=454)	3.9 (0.7)	2.9 (0.7)	7.4 (0.7)	1.9 (0.7)	1.1 (0.2)	0.2 (0.2)	0.9 (0.1)	0.2 (0.2)
2013/14 (N=626)	5.8 (0.5)	3.9 (0.5)	3.9 (0.5)	3.9 (0.5)	1.7 (0.3)	0.7 (0.3)	0.7 (0.3)	0.7 (0.3)
2014/15 (N=1,733)	6.6 (0.5)	5.1 (0.2)	4.1 (0.3)	4.8 (0.4)	2.0 (0.1)	0.2 (0.1)	0.2 (0.1)	0.2 (0.1)
2015/16 (N=852)	7.1 (0.8)	7.1 (0.8)	4.1 (1.4)	6.5 (0.5)	0.2 (0.1)	0.2 (0.1)	0.2 (0.1)	0.2 (0.1)
2016/17 (N=2,907)	10.1 (0.8)	3.8 (1.4)	7.4 (0.8)	3.8 (1.4)	0.6 (0.3)	0.6 (0.3)	0.6 (0.3)	0.6 (0.3)
2017/18 (N=2,179)	12.1 (0.3)	4.7 (0.4)	4.7 (0.6)	4.4 (0.4)	1.3 (0.2)	1.3 (0.2)	0.6 (0.3)	1.3 (0.2)
2018/19 (N=2,548)	8.3 (0.9)	8.2 (2.4)	7.8 (0.7)	6.2 (2.3)	2.4 (0.2)	1.5 (0.7)	1.7 (0.2)	1.5 (0.7)

[a] Average amino acids mismatch against all available circulating strains in respective epidemic season in ten geographical regions.

Supplementary Table 10. Prediction performance in retrospective data by geographical regions for HA of pH1N1

Country/region[a] (sample size)	Average mismatch on HA sequence (out of 566 codons) (AAs)				Average mismatch[b] on HA epitope (out of 50 sites) (AAs)			
	current-system (SD)	beth-1 (HA+NA) (SD)	LBI (HA) (SD)	beth-1 (HA) (SD)	current-system (SD)	beth-1 (HA+NA) (SD)	LBI (HA) (SD)	beth-1 (HA) (SD)
NY (N=134)	13.6 (4.3)	5.6 (2.7)	6.4 (2.6)	5.6 (2.7)	3.7 (1.0)	1.5 (0.4)	1.8 (0.7)	1.5 (0.4)
CA (N=353)	12.4 (4.4)	4.8 (3.1)	5.3 (2.6)	4.8 (3.1)	3.3 (0.9)	1.1 (0.6)	1.4 (0.8)	1.1 (0.6)
CAN (N=373)	12.8 (4.8)	5.4 (3.1)	5.7 (1.9)	5.4 (3.1)	3.5 (1.3)	1.3 (0.8)	1.7 (0.8)	1.3 (0.8)
UK (N=1,197)	12.5 (4.8)	4.8 (3.0)	5.2 (2.1)	4.8 (3.0)	3.5 (1.0)	1.2 (0.6)	1.5 (0.7)	1.2 (0.6)
GER (N=173)	12.2 (4.5)	4.4 (3.0)	5.0 (2.6)	4.4 (3.0)	3.3 (0.7)	1.0 (0.6)	1.4 (1.0)	1.0 (0.6)
FR (N=767)	12.4 (4.5)	4.5 (3.0)	5.3 (3.1)	4.5 (3.0)	3.3 (0.9)	1.1 (0.7)	1.5 (0.9)	1.1 (0.7)
HK (N=334)	12.7 (4.5)	4.9 (3.1)	5.5 (2.6)	4.9 (3.1)	3.4 (0.7)	1.1 (0.7)	1.5 (1.1)	1.1 (0.7)
SCP (N=823)	12.5 (4.3)	5.5 (3.2)	5.8 (2.8)	5.5 (3.2)	3.2 (0.6)	1.0 (0.7)	1.4 (1.1)	1.0 (0.7)
JP (N=1,292)	14 (4.2)	6.1 (2.9)	6.9 (2.9)	6.1 (2.9)	3.7 (0.7)	1.4 (0.8)	1.8 (1.1)	1.4 (0.8)
SG (N=657)	12.9 (4.5)	4.7 (2.2)	5.8 (2.8)	4.7 (2.2)	3.4 (0.8)	1.2 (0.7)	1.6 (1.0)	1.2 (0.7)
**Average (SD)**	**12.8 (4.4)**	**5.1 (2.8)**	**5.7 (2.5)**	**5.1 (2.8)**	**3.4 (0.8)**	**1.2 (0.6)**	**1.6 (0.9)**	**1.2 (0.6)**

[a] Abbreviations: NY: New York State, CA: California, CAN: Canada, UK: United Kingdom, FR: France, HK: Hong Kong SAR, SCP: South China Provinces, JP: Japan, SG: Singapore

[b] Average amino acids mismatch against all available circulating strains in epidemic seasons from 2012/13 to 2018/19 in respective geographical regions.

Supplementary Table 11. Prediction performance in retrospective data by geographical regions for NA of pH1N1

Country/region[a] (sample size)	Average number of mismatch on NA sequence (out of 469 codons) (AAs)				Average number of mismatch[b] on NA epitope (out of 30 sites) (AAs)			
	current-system (SD)	beth-1 (HA+NA) (SD)	LBI (NA) (SD)	beth-1 (NA) (SD)	current-system (SD)	beth-1 (HA+NA) (SD)	LBI (NA) (SD)	beth-1 (NA) (SD)
NY (N=134)	11.4 (4.6)	3.9 (1.7)	6.1 (1.9)	3.4 (1.6)	1.1 (0.8)	0.5 (0.6)	1.1 (1.2)	0.5 (0.6)
CA (N=353)	11.1 (4.6)	3.8 (1.8)	6 (2.0)	3.2 (1.8)	1.1 (0.9)	0.4 (0.5)	1.1 (1.2)	0.4 (0.5)
CAN (N=373)	11.7 (5.1)	4.7 (2.2)	6.4 (2.0)	4.2 (2.0)	1.4 (0.9)	0.7 (0.4)	1.3 (1.1)	0.7 (0.4)
UK (N=1,197)	11.9 (4.6)	4.5 (1.8)	6.6 (2.1)	3.9 (1.8)	1.4 (1.1)	0.7 (0.5)	1.3 (1.2)	0.7 (0.5)
GER (N=173)	11.4 (3.8)	4.3 (2.5)	6.1 (2.7)	3.8 (2.0)	1.2 (0.7)	0.5 (0.4)	1.2 (1.1)	0.5 (0.4)
FR (N=767)	11.4 (4.6)	4.1 (1.7)	6.2 (2.3)	3.6 (1.7)	1.2 (1.0)	0.5 (0.5)	1.1 (1.2)	0.5 (0.5)
HK (N=334)	11.8 (4.2)	4.7 (1.7)	6.6 (2.3)	4.2 (1.7)	1.3 (0.8)	0.6 (0.4)	1.3 (1.0)	0.6 (0.4)
SCP (N=823)	10.8 (4.0)	4.5 (0.8)	6.2 (1.9)	3.9 (1.1)	1.0 (0.8)	0.4 (0.3)	1.1 (1.0)	0.4 (0.3)
JP (N=1,292)	12.3 (4.4)	5.0 (1.5)	7.0 (2.1)	4.4 (1.3)	1.3 (0.9)	0.6 (0.4)	1.3 (1.1)	0.6 (0.4)
SG (N=657)	12.0 (4.3)	4.8 (1.5)	6.9 (1.7)	4.3 (1.5)	1.4 (1.0)	0.7 (0.5)	1.4 (1.0)	0.7 (0.5)
**Average (SD)**	**11.6 (4.4)**	**4.4 (1.6)**	**6.4 (2.1)**	**3.9 (1.5)**	**1.2 (0.8)**	**0.5 (0.4)**	**1.2 (1.1)**	**0.5 (0.4)**

[a] Abbreviations: NY: New York State, CA: California, CAN: Canada, UK: United Kingdom, FR: France, HK: Hong Kong SAR, SCP: South China Provinces, JP: Japan, SG: Singapore

[b] Average amino acids mismatch against all available circulating strains in epidemic seasons from 2012/13 to 2018/19 in respective geographical region.

Supplementary Table 12. Prediction performance in retrospective data by geographical regions for HA of H3N2

Country/region[a] (sample size)	Average number of mismatch on HA sequence (out of 566 codons) (AAs)				Average number of mismatch[b] on HA epitope (out of 131 sites) (AAs)			
	**current-system (SD)**	**beth-1 (HA+NA) (SD)**	**LBI (HA) (SD)**	**beth-1 (HA) (SD)**	**current-system (SD)**	**beth-1 (HA+NA) (SD)**	**LBI (HA) (SD)**	**beth-1 (HA) (SD)**
NY (N=1,287)	11.1 (4.8)	7.1 (3.5)	8.3 (4.0)	7.1 (3.5)	7.3 (3.0)	4.7 (2.3)	5.4 (2.8)	4.7 (2.3)
CA (N=1,011)	10.5 (4.8)	6.7 (3.5)	8.2 (4.7)	6.7 (3.5)	6.9 (3.2)	4.6 (2.5)	5.3 (3.0)	4.6 (2.5)
CAN (N=1,557)	11.2 (4.0)	8.6 (3.7)	10.5 (4.9)	8.6 (3.7)	7.0 (2.6)	5.5 (2.5)	6.4 (3.4)	5.5 (2.5)
UK (N=2,168)	11.3 (5.2)	7.6 (2.9)	9.7 (5.1)	7.6 (2.9)	7.6 (3.3)	5.3 (2.1)	6.5 (3.4)	5.3 (2.1)
GER (N=416)	9.1 (2.8)	7.5 (2.6)	9.6 (4.5)	7.5 (2.6)	6.2 (2.1)	5.1 (1.9)	6.5 (3.0)	5.1 (1.9)
FR (N=1,226)	9.5 (2.8)	7.0 (3.3)	8.9 (4.8)	7.0 (3.3)	6.2 (2.4)	4.9 (2.7)	5.8 (3.3)	4.9 (2.7)
HK (N=646)	11.1 (4.6)	7.9 (2.5)	9.2 (4.6)	7.9 (2.5)	7.5 (3.1)	5.7 (2.2)	6.3 (3.2)	5.7 (2.2)
SCP (N=1,687)	12.2 (5.4)	8.2 (2.3)	10.4 (5.1)	8.2 (2.3)	8.2 (3.4)	5.7 (1.9)	6.7 (3.0)	5.7 (1.9)
JP (N=2,394)	11.6 (5.7)	7.5 (2.9)	9.6 (5.7)	7.5 (2.9)	7.5 (3.9)	5.0 (2.3)	6.2 (3.5)	5.0 (2.3)
SG (N=1,040)	12.0 (5.4)	7.7 (2.7)	9.8 (5.5)	7.7 (2.7)	8.0 (3.7)	5.5 (2.0)	6.5 (3.3)	5.5 (2.0)
**Average (SD)**	**11.7 (5.1)**	**7.5 (2.2)**	**9.5 (4.7)**	**7.5 (2.2)**	**7.7 (3.4)**	**5.1 (1.7)**	**6.1 (2.9)**	**5.1 (1.7)**

[a] Abbreviations: NY: New York State, CA: California, CAN: Canada, UK: United Kingdom, FR: France, HK: Hong Kong SAR, SCP: South China Provinces, JP: Japan, SG: Singapore

[b] Average amino acids mismatch against all available circulating strains in epidemic seasons from 2002/03 to 2018/19 in respective geographical region.

Supplementary Table 13. Prediction performance in retrospective data by geographical regions for NA of H3N2

Country/region[a] (sample size)	Average number of mismatch on NA sequence (out of 469 codons) (AAs)				Average number of mismatch[b] on NA epitope (out of 12 sites) (AAs)			
	current-system (SD)	beth-1 (HA+NA) (SD)	LBI (NA) (SD)	beth-1 (NA) (SD)	current-system (SD)	beth-1 (HA+NA) (SD)	LBI (NA) (SD)	beth-1 (NA) (SD)
NY (N=1,129)	7.3 (2.6)	6.4 (4.4)	6.6 (3.4)	5.9 (3.8)	1.0 (0.8)	0.6 (0.7)	0.7 (0.7)	0.6 (0.7)
CA (N=904)	7.1 (2.7)	5.4 (3.5)	6.6 (3.9)	4.9 (2.5)	0.9 (0.8)	0.5 (0.7)	0.6 (0.8)	0.5 (0.7)
CAN (N=416)	7.4 (2.4)	5.6 (1.8)	6.2 (3.3)	5.2 (1.7)	0.9 (0.9)	0.5 (0.6)	0.5 (0.6)	0.5 (0.6)
UK (N=2,068)	7.5 (2.5)	5.9 (4.0)	5.0 (1.6)	5.5 (4.1)	0.9 (0.8)	0.5 (0.5)	0.5 (0.5)	0.5 (0.5)
GER (N=400)	7.3 (2.7)	5.8 (3.7)	5.9 (2.8)	4.9 (2.4)	1.0 (0.8)	0.5 (0.6)	0.6 (0.7)	0.4 (0.6)
FR (N=1,171)	7.0 (2.7)	5.2 (3.5)	5.6 (2.3)	4.5 (2.4)	0.9 (0.8)	0.5 (0.5)	0.6 (0.6)	0.4 (0.5)
HK (N=582)	7.7 (2.8)	5.5 (3.2)	7.2 (3.6)	5.1 (2.9)	1.2 (0.9)	0.6 (0.5)	0.8 (0.7)	0.5 (0.5)
SCP (N=1,475)	8.1 (3.0)	6.0 (3.8)	8.2 (4.0)	5.5 (3.5)	1.2 (1.0)	0.7 (0.7)	0.9 (0.8)	0.7 (0.7)
JP (N=2,084)	7.9 (2.9)	6.0 (3.8)	7.6 (4.7)	5.5 (3.1)	1.0 (0.9)	0.6 (0.6)	0.7 (0.7)	0.5 (0.6)
SG (N=1,032)	8.2 (2.9)	5.3 (3.5)	7.1 (3.9)	4.8 (2.7)	1.1 (0.9)	0.6 (0.5)	0.7 (0.7)	0.5 (0.5)
**Average (SD)**	**7.7 (2.5)**	**5.7 (3.0)**	**7.3 (3.6)**	**5.3 (2.3)**	**1.0 (0.8)**	**0.6 (0.5)**	**0.7 (0.6)**	**0.5 (0.5)**

[a] Abbreviations: NY: New York State, CA: California, CAN: Canada, UK: United Kingdom, FR: France, HK: Hong Kong SAR, SCP: South China Provinces, JP: Japan, SG: Singapore

[b] Average amino acids mismatch against all available circulating strains in epidemic seasons from 2002/03 to 2018/19 in respective geographical region.

Supplementary Table 14. Prediction performance for the 2019/20 season

Gene	Prediction time	Data used up to	Predicted strain	Evaluation sample size	# mismatch on full gene (SD)				# mismatch on epitopes (SD)			
					current-system	beth-1 (two-protein)	answer strain	*p*-value	current-system	beth-1 (two-protein)	answer strain	*p*-value[a]
pH1N1 HA	2019-10	2019-06	A/Hong Kong/2655/2019	1,566	11.3 (0.6)	4.7 (0.9)	4.8 (0.7)	6.1e-8	2.9 (0.2)	2.0 (0.4)	2.1 (0.3)	3.0e-4
	2022-03	2019-03	A/Hong Kong/98/2019			4.8 (0.7)		1.3e-9		2.1 (0.3)		2.4e-6
pH1N1 NA	2019-10	2019-06	A/Hong Kong/2655/2019	1,193	8.1 (0.3)	2.6 (0.6)	2.7 (0.5)	2.0e-7	1.0 (0.1)	0.1 (0.1)	0.1 (0.1)	8.9e-6
	2022-03	2019-03	A/Hong Kong/98/2019			2.7 (0.5)		5.0e-9		0.1 (0.1)		8.9e-6
H3N2 HA	2019-10	2019-06	A/Hong Kong/2678/2019	1,875	16.1 (4.3)	11.0 (3.9)	9.2 (3.8)	0.016	10.4 (3.1)	7.1 (2.2)	6.1 (2.4)	0.029
	2022-03	2019-03	A/Hong Kong/2926/2018			12.2 (3.4)		0.062		8.2 (1.7)		0.191
H3N2 NA	2019-10	2019-06	A/Hong Kong/2678/2019	1,671	9.3 (2.1)	5.5 (2.4)	5.5 (2.1)	0.001	1.8 (0.6)	0.6 (0.4)	0.6 (0.4)	4.8e-4
	2022-03	2019-03	A/Hong Kong/2926/2018			4.7 (2.3)		3.9e-4		0.6 (0.4)		4.8e-4

[a] Two-sided *p*-value is calculated using *t*-test on log mismatch between the current-system and beth-1 predictions.

Supplementary Table 15. Prediction performance by prospective validation in 2020/21 - 2022/23

Gene	Target season	Prediction time	Data used up to	Predicted strain	Evaluation sample size	# mismatch on full gene (SD)				# mismatch on epitopes (SD)			
						current-system	beth-1 (two-protein)	answer strain	*p*-value	current-system	beth-1 (two-protein)	answer strain	*p*-value[a]
pH1N1 HA	2020/21	2020-02	2019-12	A/Hong Kong/4380/2019	122	11.8 (4.5)	8.4 (2.5)	4.9 (6.0)	7.6e-4	3.8 (1.2)	2.7 (1.1)	1.3 (2.0)	3.7e-9
	2021/22	2021-02	2021-01	A/NAGASAKI/8/2020	1,358	10.1 (2.8)	10.2 (7.2)	8.2 (8.1)	0.545	3.6 (1.3)	3.3 (2.5)	2.3 (2.5)	< 2.2e-16
	2022/23	2022-02	2022-01	A/Bangladesh/3210910005/2021	1,949	10.7 (1.9)	4.1 (1.8)	4.1 (1.9)	1.3e-5	3.0 (0.7)	1.0 (0.7)	1.0 (0.7)	3.1e-5
pH1N1 NA	2020/21	2020-02	2019-12	A/Hong Kong/4380/2019	111	5.9 (2.0)	2.8 (3.0)	2.4 (3.6)	< 2.2e-16	0.2 (0.5)	0.2 (0.5)	0.2 (0.5)	1
	2021/22	2021-02	2021-01	A/NAGASAKI/8/2020	1,360	3.3 (2.1)	8.3 (2.0)	3.2 (2.8)	< 2.2e-16	0.2 (0.5)	0.2 (0.5)	0.2 (0.5)	1
	2022/23	2022-02	2022-01	A/Bangladesh/3210910005/2021	1,864	4.1 (0.4)	2.5 (0.9)	2.5 (1.5)	4.5e-5	0.2 (0.2)	0.2 (0.2)	0.2 (0.2)	1
H3N2 HA	2020/21	2020-02	2019-12	A/Hong Kong/4435/2019	493	19.3 (3.6)	18.9 (4.3)	5.7 (4.5)	0.031	13.1 (2.6)	13.6 (3.4)	4.2 (3.3)	0.553
	2021/22	2021-02	2021-01	A/Bangladesh/0004/2020	15,476	13.1 (2.5)	5.7 (2.3)	4.2 (4.0)	< 2.2e-16	10.8 (1.9)	4.2 (1.6)	2.9 (2.7)	< 2.2e-16
	2022/23	2022-02	2022-01	A/Arsal/5773/2021	5,592	9.8 (1.8)	9.1 (2.1)	7.7 (3.1)	0.327	6.3 (1.5)	5.9 (1.7)	5.0 (2.2)	0.485
H3N2 NA	2020/21	2020-02	2019-12	A/Hong Kong/4435/2019	469	6.4 (1.4)	5.4 (1.4)	2.6 (1.8)	3.9e-4	1.2 (0.4)	0.2 (0.4)	1.2 (0.5)	< 2.2e-16
	2021/22	2021-02	2021-01	A/Bangladesh/0004/2020	15,074	3.1 (1.5)	2.3 (1.6)	2.3 (2.0)	< 2.2e-16	1.3 (0.9)	0.2 (0.5)	0.2 (0.5)	< 2.2e-16
	2022/23	2022-02	2022-01	A/Arsal/5773/2021	5,342	5.0 (0.9)	3.9 (0.9)	3.9 (1.0)	0.025	0.4 (0.3)	0.4 (0.3)	0.4 (0.4)	1

[a] Two-sided *p*-value is calculated using *t*-test on log mismatch between the current-system and beth-1 predictions.

Supplementary Table 16. Summary table of fitted parameters

	**pH1N1 HA**				**pH1N1 NA**				**H3N2 HA**				**H3N2 NA**			
	HK[a]	SG	IN	TH	HK	SG	IN	TH	HK	SG	IN	TH	HK	SG	IN	TH
p-value[b] of g-measure	0.013*	0.001**	0.034*	0.004**	0.056	0.0004***	0.025	0.006**	0.087	0.002**	0.023*	0.002**	0.012*	0.001**	0.050	0.160
p-value[b] of the F-statistic	0.044*	0.001**	0.049*	0.034*	0.181	0.0007***	0.065	0.046*	0.196	0.001**	0.077	0.010	0.042*	0.0007***	0.146	0.395
multiple R-squared	0.669	0.898	0.703	0.693	0.599	0.943	0.727	0.759	0.457	0.904	0.709	0.738	0.674	0.918	0.630	0.337
region specific (θ, h)[c]	(0.7,0)	(0.8,2)	(0.7,4)	(0.9,0)	(0.6,6)	(0.5,6)	(0.6,0)	(0.2,8)	(0.8,5)	(0.9,2)	(0.6,7)	(0.5,2)	(0.9,6)	(0.5,7)	(0.3,8)	(0.5,4)
median transition time τ (month)	21.8 (IQR 17.4-27.1)				13.3 (IQR 9.6-20.3)				17.4 (IQR 8.9-25.3)				11.8 (IQR 4.7-21.7)			
mean transition time τ (month)	22.7 (SD 8.5)				16.0 (SD 10.3)				20.3 (SD 15.6)				14.6 (SD 12.0)			

[a] HK: Hong Kong SAR, SG: Singapore; IN: India; TH: Thailand

[b] Two-sided *p*-value estimated by multiple linear regression.

[c] Region-specific θ is used to calculate transition time, and it is estimated by fitting the linear regression model of the g-measure and covariates including mean temperature, absolute humidity and season against annual sero-positivity rate. The annual mean temperature (centigrade) and mean absolute humidity (g/m^3) were retrieved from Hong Kong Observatory via https://www.hko.gov.hk/contentc.htm for Hong Kong SAR, and Weather Underground via https://www.wunderground.com/ for Singapore, India and Thailand. The yearly number of positive cases and specimens were retrieved from Hong Kong Center for Health Protection (HKHCP) via https://www.chp.gov.hk/en/resources/29/304.html for Hong Kong SAR, and FluNet of Global Influenza Surveillance and Response System (GISRS) via https://www.who.int/tools/flunet for Singapore, India and Thailand. The annual sero-positivity rate is calculated as number of positive cases divided by number of specimens. These epidemiology data are provided in https://github.com/mwanglab/beth-1/tree/main/epidemic_data.

Supplementary Table 17. Detailed information of the reagents and cell lines used in this study.

Reagents or resources	Source	Identifier
Minimum essential medium (MEM)	Thermo Fisher Scientific	31095029
Penicillin-Streptomycin (PS)	Thermo Fisher Scientific	15140122
Trypsin-EDTA	Thermo Fisher Scientific	25200056
Fetal Bovine Serum (FBS)	Thermo Fisher Scientific	26140079
HEPES	Thermo Fisher Scientific	15630080
N-p-Tosyl-L-phenylalanine chloromethyl ketone (TPCK)	Sigma-Aldrich	4370285
HA and NA gene	Sangon Technology	N/A
PR8 8 plasmids	This study	N/A
phw2000 vector	[4]	N/A
TransIT®-LT1 Transfection Reagent	Mirus Bio	MIR 2306
AddaVax™	InvivoGen	vac-adx-10
**Cell lines**	**Source**	**Identifier**
HEK 293T	N/A	N/A
Humanized MDCK cells	[5]	N/A

Supplementary references:

1 Wang, M. H. et al. Characterization of key amino acid substitutions and dynamics of the influenza virus H3N2 hemagglutinin. The Journal of infection 83, 671-677, doi:10.1016/j.jinf.2021.09.026 (2021).

2 Cao, L. et al. In silico prediction of influenza vaccine effectiveness by sequence analysis. Vaccine 39, 1030-1034, doi:10.1016/j.vaccine.2021.01.006 (2021).

3 Cao, L. et al. Improving the prediction of influenza vaccine effectiveness by refined genetic distance measure. medRxiv, 2023.2002. 2014.23285900 (2023).

4 Liang, W. et al. Egg-adaptive mutations of human influenza H3N2 virus are contingent on natural evolution. PLoS Pathog 18, e1010875, doi:10.1371/journal.ppat.1010875 (2022).

5 Takada, K. et al. A humanized MDCK cell line for the efficient isolation and propagation of human influenza viruses. Nature microbiology 4, 1268-1273, doi:10.1038/s41564-019-0433-6 (2019).
