# Supplementary material for: Predictive evolutionary modelling for influenza virus by site-based dynamics of mutations
Source: Nat Commun. 2024 Mar 21;15:2546. doi: 10.1038/s41467-024-46918-0 (PMC10958014; doi:10.1038/s41467-024-46918-0)
Supplement: Supplementary file 3 — Reporting Summary [file 41467_2024_46918_MOESM3_ESM.pdf]

nature portfolio

Corresponding author(s): Maggie Haitian Wang, Chris Ka Pun Mok

Last updated by author(s): Mar 6, 2024

Reporting Summary

Nature Portfolio wishes to improve the reproducibility of the work that we publish. This form provides structure for consistency and transparency in reporting. For further information on Nature Portfolio policies, see our Editorial Policies and the Editorial Policy Checklist.

Statistics

For all statistical analyses, confirm that the following items are present in the figure legend, table legend, main text, or Methods section.

n/a	Confirmed	
☐	☒	The exact sample size (n) for each experimental group/condition, given as a discrete number and unit of measurement
☒	☐	A statement on whether measurements were taken from distinct samples or whether the same sample was measured repeatedly
☐	☒	The statistical test(s) used AND whether they are one- or two-sided<br>*Only common tests should be described solely by name; describe more complex techniques in the Methods section.*
☒	☐	A description of all covariates tested
☒	☐	A description of any assumptions or corrections, such as tests of normality and adjustment for multiple comparisons
☐	☒	A full description of the statistical parameters including central tendency (e.g. means) or other basic estimates (e.g. regression coefficient) AND variation (e.g. standard deviation) or associated estimates of uncertainty (e.g. confidence intervals)
☐	☒	For null hypothesis testing, the test statistic (e.g. F, t, r) with confidence intervals, effect sizes, degrees of freedom and P value noted<br>*Give P values as exact values whenever suitable.*
☒	☐	For Bayesian analysis, information on the choice of priors and Markov chain Monte Carlo settings
☒	☐	For hierarchical and complex designs, identification of the appropriate level for tests and full reporting of outcomes
☒	☐	Estimates of effect sizes (e.g. Cohen's d, Pearson's r), indicating how they were calculated

Our web collection on statistics for biologists contains articles on many of the points above.

Software and code

Policy information about availability of computer code

Data collection	No software were used for data collection.
Data analysis	The study used custom algorithms that are made available to editors and reviewers in GitHub at https://github.com/mwanglab/beth-1. R version 4.1.3 was used to analyze data, and the R packages used in analysis includes Biostring (2.64.1), lubridate (1.8.0), plyr (1.8.7) and stringr (1.4.0).

For manuscripts utilizing custom algorithms or software that are central to the research but not yet described in published literature, software must be made available to editors and reviewers. We strongly encourage code deposition in a community repository (e.g. GitHub). See the Nature Portfolio guidelines for submitting code & software for further information.

Data

Policy information about availability of data

All manuscripts must include a data availability statement. This statement should provide the following information, where applicable:

- Accession codes, unique identifiers, or web links for publicly available datasets
- A description of any restrictions on data availability
- For clinical datasets or third party data, please ensure that the statement adheres to our policy

All data used in this study are publicly available. Viral sequence data were downloaded from the global initiative on sharing all influenza data (GISAID) at http://

platform.gisaid.org/ and the accession numbers were provided in the online supplementary acknowledgment table (https://github.com/mwanglab/beth-1/tree/main/acknowledgement_table).

Research involving human participants, their data, or biological material

Policy information about studies with human participants or human data. See also policy information about sex, gender (identity/presentation), and sexual orientation and race, ethnicity and racism.

Reporting on sex and gender	This information has not been collected.
Reporting on race, ethnicity, or other socially relevant groupings	This information has not been collected.
Population characteristics	This information has not been collected.
Recruitment	The biological samples are lab confirmed influenza positive cases admitted in the Prince of Wales Hospital. The study samples is randomly drawn from the pool of influenza positive cases.
Ethics oversight	the Joint Chinese University of Hong Kong – New Territories East Cluster Clinical Research Ethics Committee

Note that full information on the approval of the study protocol must also be provided in the manuscript.

Field-specific reporting

Please select the one below that is the best fit for your research. If you are not sure, read the appropriate sections before making your selection.

☐ Life sciences ☐ Behavioural & social sciences ☒ Ecological, evolutionary & environmental sciences

For a reference copy of the document with all sections, see nature.com/documents/nr-reporting-summary-flat.pdf

Ecological, evolutionary & environmental sciences study design

All studies must disclose on these points even when the disclosure is negative.

Study description	This study analyzed serial cross-sectional data of influenza virus genetic sequences from public database, and used virus isolated from local hospital to validate the model finding.
Research sample	Virus were isolated from Nasopharyngeal aspirate samples of lab confirmed influenza positive cases admitted to the Prince of Wales Hospital, Hong Kong SAR. To select representative strains in Hong Kong in the 2019-20 season, 20 nasal swabs positive to influenza A virus detected by PCR method were randomly selected for further serotyping into H1 and H3 by PCR using specific primers.
Sampling strategy	Ten samples were randomly selected for each influenza A subtype in epidemic season 2019/20, and one pH1N1 virus and one H3N2 virus from these isolated were randomly selected for animal experiments. Phylogenetic analysis showed that the isolates belonged to the same cluster, of which HA shared >98% amino acid similarity for pH1N1 and >96% for H3N2.
Data collection	The clinical samples were collected as a part of the routine clinical management. Nasal pharyngeal samples of patients suspected with respiratory infection in different clinical departments of the Prince of Wales Hospital are collected and sent to the Microbiology Department for testing various infectious diseases including influenza. HA sequences were obtained from Sanger sequencing and recorded in fasta format.
Timing and spatial scale	The data were collected and generated between 2019-09 to 2020-02 in the Prince of Wales Hospital and the University of Hong Kong.
Data exclusions	No data was excluded.
Reproducibility	Ten mice representing biological replicates were immunized successfully with response against inoculums, and included for downstream analysis in this study.
Randomization	Randomization is not applicable for the animal experiment in this study, and results from all mice were used in analysis.
Blinding	In this study, information including patients gender and age was not collected, and thus did not cause bias on analysis of viral isolation or serotyping.

Did the study involve field work? ☐ Yes ☒ No

Reporting for specific materials, systems and methods

We require information from authors about some types of materials, experimental systems and methods used in many studies. Here, indicate whether each material, system or method listed is relevant to your study. If you are not sure if a list item applies to your research, read the appropriate section before selecting a response.

Materials & experimental systems

n/a	Involved in the study
☒	☐ Antibodies
☐	☒ Eukaryotic cell lines
☒	☐ Palaeontology and archaeology
☐	☒ Animals and other organisms
☒	☐ Clinical data
☒	☐ Dual use research of concern
☒	☐ Plants

Methods

n/a	Involved in the study
☒	☐ ChIP-seq
☒	☐ Flow cytometry
☒	☐ MRI-based neuroimaging

Eukaryotic cell lines

Policy information about cell lines and Sex and Gender in Research

Cell line source(s)	Human embryonic kidney (HEK) 293T cells were purchased from ATCC. Humanized Madin-Darby canine kidney (hMDCK) cells were provided by Yoshihiro Kawaoka from the University of Tokyo.
Authentication	Humanized Madin-Darby canine kidney (hMDCK) cell line has been authenticated by Professor Yoshihiro Kawaoka from the University of Tokyo.
Mycoplasma contamination	All cell lines used in this study have been constantly tested negative for mycoplasma.
Commonly misidentified lines (See ICLAC register)	Nil

Animals and other research organisms

Policy information about studies involving animals; ARRIVE guidelines recommended for reporting animal research, and Sex and Gender in Research

Laboratory animals	Balb/c mice. 18-22 degrees, 40-60% humidity. Housing condition was 12h light/12h dark cycle.
Wild animals	Nil
Reporting on sex	80 female balb/c mice were used in this study. We chose female mice for our experiment as they are less aggressive than male which cause less danger on biosafety. While our study aimed to study the matching of antigenicity of influenza virus, the gender of the animal should not be a crucial factor to influence our results as well as the conclusion.
Field-collected samples	Nil
Ethics oversight	Teaching and Research Committee on the Use of Live Animals (CULATR 5598-20) of the University of Hong Kong

Note that full information on the approval of the study protocol must also be provided in the manuscript.

Plants

Seed stocks	Not applicable.
Novel plant genotypes	Not applicable.
Authentication	Not applicable.
